# Drosophila Models Rediscovered with Super-Resolution Microscopy

**DOI:** 10.3390/cells10081924

**Published:** 2021-07-29

**Authors:** Szilárd Szikora, Péter Görög, Csaba Kozma, József Mihály

**Affiliations:** 1 Institute of Genetics, Biological Research Centre, Temesvári krt. 62, H-6726 Szeged, Hungary; gorog.peter@brc.hu; 2 Doctoral School of Multidisciplinary Medical Science, Faculty of Medicine, University of Szeged, H-6725 Szeged, Hungary; 3 Foundation for the Future of Biomedical Sciences in Szeged, Szeged Scientists Academy, Pálfy u. 52/d, H-6725 Szeged, Hungary; kozma.csaba7122@gmail.com; 4 Department of Genetics, University of Szeged, H-6726 Szeged, Hungary

**Keywords:** nanoscopy, super-resolution microscopy, SIM, STED, SMLM, *Drosophila*, active zone, centrosome, sarcomere

## Abstract

With the advent of super-resolution microscopy, we gained a powerful toolbox to bridge the gap between the cellular- and molecular-level analysis of living organisms. Although nanoscopy is broadly applicable, classical model organisms, such as fruit flies, worms and mice, remained the leading subjects because combining the strength of sophisticated genetics, biochemistry and electrophysiology with the unparalleled resolution provided by super-resolution imaging appears as one of the most efficient approaches to understanding the basic cell biological questions and the molecular complexity of life. Here, we summarize the major nanoscopic techniques and illustrate how these approaches were used in *Drosophila* model systems to revisit a series of well-known cell biological phenomena. These investigations clearly demonstrate that instead of simply achieving an improvement in image quality, nanoscopy goes far beyond with its immense potential to discover novel structural and mechanistic aspects. With the examples of synaptic active zones, centrosomes and sarcomeres, we will explain the instrumental role of super-resolution imaging pioneered in *Drosophila* in understanding fundamental subcellular constituents.

## 1. Introduction

Optical microscopy uses visible light and a series of lenses to image and magnify biological specimens. In recent decades, fluorescent microscopy has become the most popular tool of cell and developmental biologists, since fluorescent labeling offers high molecular specificity, and due to its low invasiveness, it has become the leading method to study living cells, tissues or organisms. In the early days, only widefield applications were available, but with later technological advances, every aspect of microscopy was improved tremendously, resulting in the development of specialized microscopes, such as optical sectioning confocal microscopes, spinning disk confocal microscopes, light sheets, TIRF and multi-photon microscopic tools.

**Figure 1:**
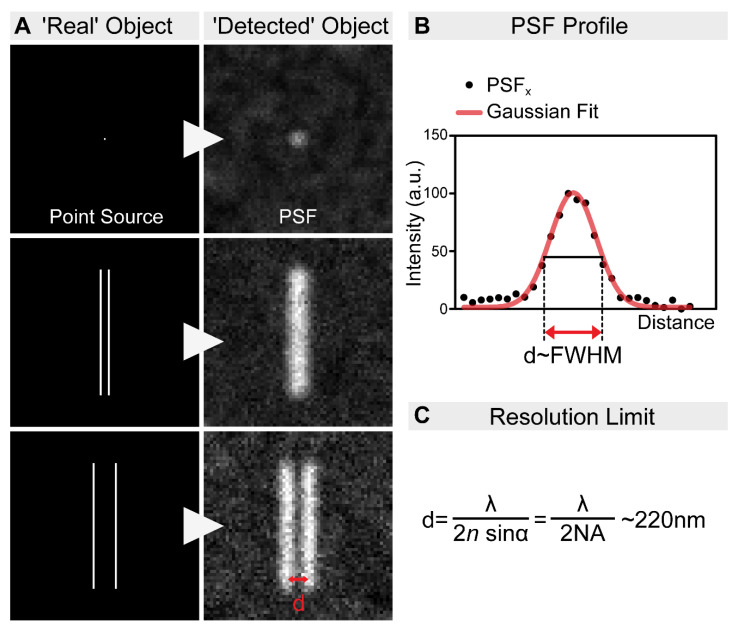
The resolution limit of optical microscopes. (**A**) In practice, even an extremely small point source of light (e.g., a single fluorophore) is still imaged as a spot of finite volume, known as the point spread function (PSF). Therefore, when two objects are close to each other, it is impossible to distinguish them. In order to discriminate them as two individual objects, they need to be separated by an adequate distance (d). This distance, by definition, is the resolution of the microscope. (**B**) The resolution of an optical system can be estimated by measuring the full-width at half-maximum (FWHM) of the PSF. (**C**) The spatial resolution of an optical imaging system is limited by the diffraction of the light, which was first described in 1873 by Ernst Abbe. This resolution limit in microscopy is calculated by the following equation: λ/2NA, where λ is the wavelength of the light, and NA is the numerical aperture of the objective. Accordingly, even in the most favorable condition, with a high NA objective and the lowest possible wavelength, the resolution will never be higher than ~220 nm.

Today’s scientists wish to understand the biological processes at a molecular level. Fluorescent microscopy, allowing the labeling of biomolecules with high specificity by the use of antibodies or by tagging with fluorescent proteins, appeared as a great tool for such an aim. However, the spatial resolution of fluorescent microscopes is limited by the wavelength of light at around ~220 nm (Figure 1). While this resolution is sufficient to visualize cellular organelles, it is not sufficient to resolve the molecular organization of these complexes. In parallel with the development of the optical approaches, multiple tools have been invented to overcome this obstacle. For example, electron microscopy (EM) allows ultrastructural-level analyses of biological structures, and the detection of specific molecules can also be accomplished by immunogold labeling. However, achieving high-density immunogold labeling is challenging, and it is far from being efficient. Scanning probe and nearfield optical scanning microscopy also offer outstanding resolution, but, unfortunately, they are largely surface-bound and not able to image the intact cellular interior, and hence their popularity is limited. In the end, the main goal remained the same: to go under the diffraction limit by using visible light. The breakthrough came from revisiting the principles of fluorescence microscopy, and that is what we call fluorescence nanoscopy today (also often referred to as super-resolution microscopy (SRM)). By bridging the gap between conventional light microscopy and structural biology, the founders of these new concepts were awarded the Nobel Prize in Chemistry in 2014. In this review, we summarize the principles of the main nanoscopic approaches, and their impact on understanding the molecular organization of several highly conserved cellular machineries by using *Drosophila* models.

## 2. Overview of the Nanoscopic Methods

The resolution or diffraction limit of optical microscopes defines the minimal distance between two points that can be resolved, and the minimal size of the focal point. This means that the fluorophores in the focal point are inseparable, as these are excited at the same time and emit light together (Figure 1). The various super-resolution methods use different approaches to circumvent this limitation, and below, we summarize the main features of these techniques.

### 2.1. Structured Illumination Microscopy—SIM

**Figure 2:**
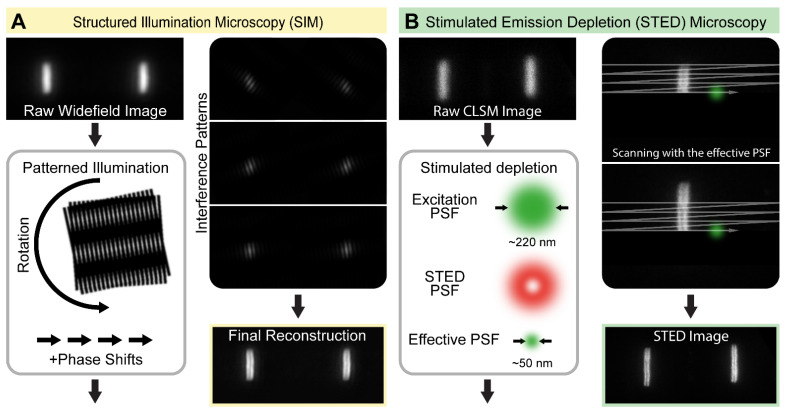
The concept of structured illumination microscopy (SIM) and stimulated emission depletion (STED) microscopy. (**A**) SIM is a widefield microscopic approach that relies on generating interference patterns (moiré fringes) in the plane of imaging through illuminating the samples with a periodic pattern. Images are acquired in multiple orientations and angles, and the high-resolution image is reconstructed computationally. (**B**) STED microscopy is a point scanning approach that relies on generating a subdiffraction-sized focal point in the sample plane. It is achieved by inducing a stimulated emission with a doughnut-shaped STED beam that is spatially overlaid with a regular excitation beam. The effective PSF, and therefore the resolution, is tuned by the intensity of the depleting STED laser. The sample is scanned by the co-aligned beams, which provides an image with a significantly improved resolution.

SIM is a widefield approach that uses patterned illumination to generate information containing interferences [1]. Due to the diffraction limit, normally, the high-frequency (high-resolution) information is lost. During a typical SIM image acquisition, the sample is illuminated by a periodic pattern generated by two interfering coherent excitation beams in the focal plane of the objective lens. The illumination pattern is projected to the focal plane in multiple phases and angles. The recorded images contain interference patterns (moiré fringes), which emerge as the fine pattern of the structured excitation light, superimposed with the fine but conventionally unresolvable cellular structures. The high-frequency information can be extracted computationally from the known illumination pattern and from the observed moiré fringes (Figure 2A). Since the illuminating standing wave pattern is also limited by the diffraction limit, the lateral resolution can only be improved by a factor of about 2 (~100 nm). However, the key advantages of SIM are the lack of the need for special sample preparation, the compatibility with conventional fluorophores and that multicolor imaging is readily available.

While SIM is a widefield approach, its 3D resolution can be improved significantly by adding a third beam which creates an interference pattern in the axial plain [2,3] or by combining it with lattice light sheet microscopy [4]. The 3D SIM method uses grating, which creates three mutually coherent light beams to attain different illumination patterns in the axial and lateral planes to achieve a resolution of ~100 nm laterally and ~300 nm axially [3]. The lattice light sheet setup eliminates the out-of-focus fluorescence emission, which would otherwise overwhelm the weak nonlinear harmonic signals, and also increases the axial resolution while reducing photobleaching and phototoxicity [4].

SIM is also suited for live imaging as it uses a relatively low intensity light compared to other super-resolution techniques, meaning photo-damage can be minimized, and the image acquisition is relatively fast. The acquisition time is mostly limited by illumination pattern modulation, and the speed and sensitivity of the camera. Current custom-built 2D setups are able to achieve video rates (25 reconstructed frames per second or more) while simultaneously imaging with two excitation wavelengths [5]. The spatial resolution improvement with the linear variations of SIM is moderate (only twofold) compared to the other nanoscopic approaches; however, it can be further improved by exploiting the nonlinear response of the fluorophores. Nonlinear variations of SIM (PA NL-SIM), which use photoswitchable proteins and patterned activation, can achieve a spatial resolution of ~50 nm in biological specimens [6,7].

SIM microscopy played an essential role in resolving the layered structure of *Drosophila* centrosomes [8,9], and in determining the sarcomeric localization of the nebulin repeat Lasp protein in the flight muscles of *Drosophila* [10]. For a more detailed summary on SIM microscopy and its biological applications, see: [11].

### 2.2. Stimulated Emission Depletion—STED

The principle of STED microscopy was first described in 1994 by Stefan W. Hell [12]. This approach overcomes the diffraction limit by using a focused excitation laser and a second de-excitation or STED laser with a doughnut-shaped beam profile in the focal plane [12,13]. The STED laser in the far-red part of the spectrum is able to deplete the excited state in an intensity-dependent manner, before fluorescence even takes place. The excitation laser will excite every fluorophore in the diffraction-limited focal point. The STED laser beam is superimposed with the excitation beam, and hence only fluorophores in the middle of the doughnut shape are able to emit light where the depletion laser intensity is low/zero, while the fluorophores are turned off at the outer rim of the excitation spot. As a result, the size of the effective excitation spot decreases, and scanning the whole sample with the co-aligned laser setup will result an image where the spatial resolution is improved significantly (Figure 2B).

In theory, the effective spot size can be so small that only very few fluorophores are able to emit light, although in reality, it is affected by many factors [12,14]. While a stronger depletion laser intensity produces a narrower emission zone, resulting in a higher resolution, it might also lead to phototoxicity and photobleaching [15]. In addition, the depletion curve is not rectangular, and with the higher laser intensity, there is a loss in signal strength [12]. In practice, depending on the sample and the quality of the label, STED is usually able to achieve a lateral resolution in the 30–50 nm range. Multicolor STED imaging is also possible with different methods and microscope setups. One of the setups uses conventional dyes and multiple STED lasers, one for each label. The limitation of this configuration is that the blue-shifted STED laser tends to cause strong excitation and photobleaching in the most red-emitting dye [16]. The other approach depends on the combination of carefully selected dyes, where the emission spectra of the dyes overlap, but one of them will have a long Stokes shift. This combination allows simultaneous multicolor STED recording with only one STED laser [17].

Since STED is a confocal-based system, it is ideal to study thicker specimens in 3D. Besides the lateral resolution improvement, multiple approaches have been developed to create a de-excitation beam that restricts the fluorescence in the axial axis over and under the fluorescence free zone, thereby allowing super-resolved 3D imaging. (i) Using a vortex plate in combination with an additional phase plate that, upon interference, inhibits fluorescence along the Z axis can achieve fluorescence restriction along all spatial directions [18]. (ii) An isotropic 3D focal spot (isoSTED) can also be attained with an opposing objective lens (4Pi arrangement) and can lead to a resolution of ~30 nm [19,20]. (iii) In combination with single-plane illumination microscopy (SPIM), STED is able to achieve a good axial resolution with more than 100 µm penetration depths [21].

Since STED is a point scanning method, its temporal resolution mostly depends on the scanning speed and size of the field of view. Therefore, to achieve a high temporal resolution, a small field of view and a moderate spatial resolution are necessary. As STED uses a high laser intensity, which may lead to photobleaching or phototoxicity, this makes it less appealing for live imaging. However, due to its fast scanning ability, video-rate (28 frame/s) STED imaging was demonstrated to reach a resolution of 68 nm within a 1.8 by 2.5 µm area [22]. This was further improved by using ultrafast electro-optical scanning to achieve a framerate of >1000/s in a 250 by 320 pixel area, with a resolution of 70 nm [23].

STED microscopy has been used extensively to study the molecular arrangement of *Drosophila* synapses [24,25], as discussed below in the section titled ‘Chemical Synapses’. For a more extended summary on STED microscopy and its biological applications, see: [26].

### 2.3. SMLM Methods—PALM, STORM, dSTORM, GSD, PAINT, MINFLUX

Due to the diffraction limit, even a point source of light (i.e., a single fluorescent molecule) is still imaged as a blurry spot, known as the point spread function (PSF). However, the position of this point source can be precisely determined (localized) by fitting a 2D Gaussian function with the PSF [27]. In this case, the localization precision is limited by the accuracy of fitting which is primarily dependent on the number of photons collected. Thus, the position of a sufficiently bright molecule can be determined with sub-nanometer precision [28] with a sensitive camera. The concept of single-molecule imaging was first demonstrated in 1989 [29] and has mostly been used in in vitro TIRF measurements, in a technique generally known as single-particle tracking. The most notable examples demonstrate the processive hand-over-hand walking of myosin molecules along actin filaments [30].

**Figure 3:**
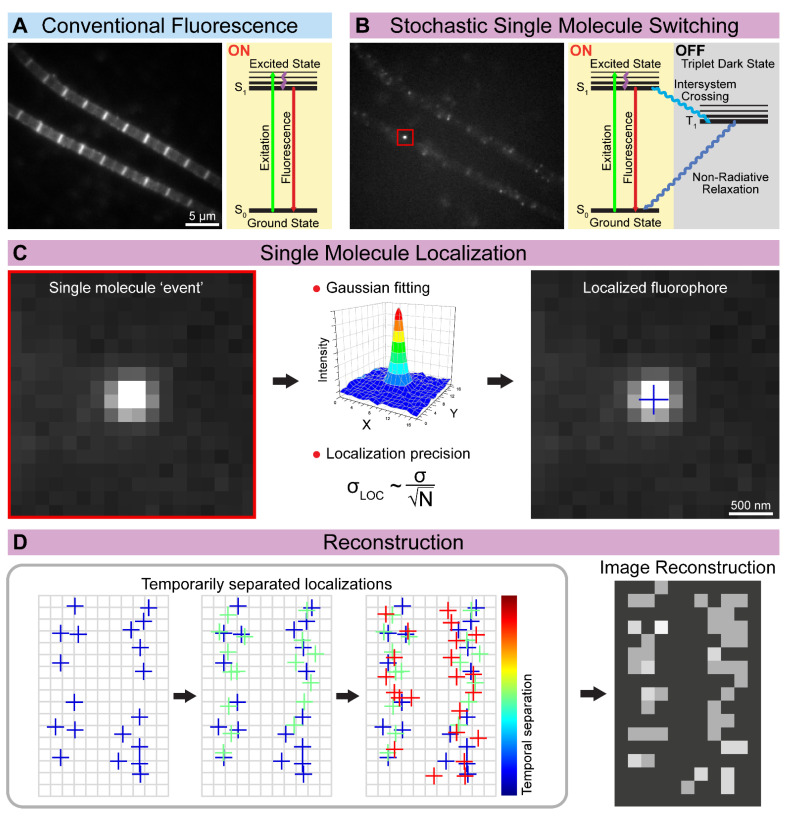
The concept of single-molecule localization microscopy (SMLM). (**A**) The conventional widefield approaches excite all the fluorescent molecules simultaneously, while (**B**) the SMLM approaches stochastically switch on only a small batch of fluorophores at a given time. (**C**) The position of the sparsely localized individual fluorescent molecules is determined based on their diffraction-limited image. The centroid position is estimated by 2D Gaussian fitting, and theoretically, the precision (σ_LOC_) is only limited by the number of photons (N) detected. (**D**) The spatially separated single-molecule events are recorded over time, and their determined position is used to reconstruct the final high-resolution image.

Since single-molecule imaging relies on spatially separated molecules, the concept cannot simply be translated to densely labeled biological specimens where the focal spots of molecules overlap. Single-molecule localization microscopy (SMLM) imaging techniques overcome this obstacle by allowing only a small subset of spatially separated molecules to stay in their fluorescent on state at a given time. By stochastically switching between the on (light) and off (dark) states of the fluorophores, SMLM achieves the temporal separation of the otherwise overlapping signals of the fluorescent molecules. As a primary output, SMLM generates millions of localization coordinates with associated uncertainties, and the super-resolved images are reconstructed from this dataset (Figure 3). The achievable lateral resolution is around 20 nm in an ideal case.

The currently applied SMLM methods include numerous approaches, which mainly differ in the method of fluorophore switching/detection: photoactivated localization microscopy (PALM) [31], stochastic optical reconstruction microscopy (STORM) [32], direct STORM (dSTORM) [33], ground state depletion (GSD) [34,35], point accumulation for imaging in nanoscale topography (PAINT) [36] and minimal photon flux (MINFLUX) [37]. PALM uses photoswitchable fluorescent proteins, such as photoactivatable green fluorescent protein, which are activated and excited. Every cycle starts with a low-wavelength laser pulse, which is able to turn a few fluorophores into their active state stochastically. These activated fluorophores can then be excited, and their localization can be determined before becoming photobleached and starting a new activation cycle [31,38]. The original STORM concept uses pairs of fluorescent dyes, an activator (Cy3) and a reversible photoswitchable reporter (Cy5) [32], and two laser lines. By a strong red laser pulse, every fluorophore is switched into a reversible dark state, and in every imaging cycle, a subset of molecules transitions into the on state by a weak green laser activation, and then it is excited, recorded and switched off again by the red laser. Fluorophores can be cycled through and recorded hundreds of times before photobleaching. Direct STORM (dSTORM) is an alternative variant of STORM which only uses a reporter dye (usually cyanin dyes: Cy5/Alexa 647) that can be reversibly switched between the two states with high efficiency by alternating laser beams, without the activator dye [33]. Later, an improvement was made, and the current dSTORM and GSD approaches use an oxygen-scavenging buffer system which promotes the blinking behavior upon red laser illumination and does not require an additional laser for the activation [39]. PAINT is based on targeting biomolecules by fluorescent probes diffusing in the solution. Each probe that hits the object and becomes immobilized appears as a diffraction-limited spot and can be located with high precision. The label then shortly dissociates from the object or becomes photobleached.

Most of the techniques mentioned above are highly light-sensitive, and one needs to find a balance between the collected number of photons and photobleaching. One of the newest concepts, MINFLUX, is able to overcome this limitation and achieve a higher resolution than any other super-resolution techniques [37,40] by combining the strength of SMLM and STED microscopy. MINFLUX switches fluorophores individually, as with SMLM, but determines their localization by using a doughnut-shaped scanning beam; however, in contrast to STED, this is an excitation beam with an intensity minimum at its center. The fluorescent molecules do not emit light when their positions coincide with the zero-intensity center of the beam, and their position can be determined by the local emission minima with molecular-scale precision (<5 nm) with a significantly lower photon count.

To gain 3D information with SMLM microscopes, which are usually widefield or TIRF-based systems, an additional setup is required which either simultaneously records multiple images of the same emitter using different beam paths or engineers the 3D shape of the PSF. A frequently used approach is to encode the axial position of the fluorophore by placing a cylindrical lens in the imaging path [41,42]. The introduced astigmatism generates elliptically shaped emitter images whose ellipticity and orientation depend on the axial particle position. The axial position can therefore be determined by analyzing the shape of the particle image, and the lateral position is obtained from the image center. If a fluorophore is in the focal plane, its PSF is round; however, when the fluorophore is above the focal plane, the PSF image appears ellipsoidal with its long axis along x. Conversely, when the fluorophore is below the focal plane, the image appears ellipsoidal with its long axis along y. After a 2D elliptical Gaussian function fitting, not only the x and y coordinates but also the z coordinate of the molecules can be determined [41], with a precision of 50–60 nm. For determining the 3D position of the fluorophores, further methods emerged, including phase-shifting interferometry [43], bifocal plane imaging [44,45], double-helix imaging [46] or Airy beam detection [47].

SMLM techniques usually need a relatively long acquisition time (measured in minutes) to record enough single-molecule events to achieve a high localization density and high resolution [48]. As a consequence, there is a trade-off between the spatial resolution and the temporal resolution with STORM and PALM. Yet, PALM was able to achieve a spatial resolution of 60 nm and a 25 s/frame temporal resolution with Eos fluorescent protein labeling [48]. Altogether, with brighter and faster-switching fluorophores, imaging can be much faster, as it was demonstrated for STORM, where it was possible to achieve a sub-second acquisition time and 20–30 nm spatial resolution with fast-switching organic dyes and an sCMOS camera [49].

SMLM microscopy combined with structure averaging was fundamental in resolving the nanoscale organization of *Drosophila* flight muscle sarcomeres [50]. For a more detailed summary on SLML microscopy and its biological applications, see: [51].

### 2.4. Expansion Microscopy 

In addition to the best known SIM, STED and SMLM methods, expansion microscopy (ExM) offers a unique way to overcome the resolution limit by expanding the specimen to allow the imaging of otherwise subdiffraction-sized biological structures [52]. The imaging protocol starts with embedding the fluorescently labeled sample in a swellable hydrogel with a non-invasive methodology. Then, the fluorescent markers (or other biomolecules) are anchored to the polymer network by chemical crosslinking, which is followed by homogenization (e.g., by proteolysis) of the specimen. This step prevents the distortion of the gel during expansion. The labeling probes survive this process and remain attached to the gel. By the addition of water, the gel is expanded in an isotropic way, which pulls apart the specimen, and the anchored fluorescent markers become separated from each other. Then, the sample can be observed with a conventional fluorescent microscope. Due to the physical magnification of the specimen, the effective resolution increases both laterally and axially. However, all the measured distances have to be divided by the calculated expansion factor to obtain the real values. ExM offers multicolor ‘super-resolution’ imaging with conventional diffraction-limited fluorescent microscopes. The achievable resolutions (~70 nm in the lateral direction, and ~200 nm in the axial direction) are comparable to other super-resolution methods [52] and can be further improved by combining ExM with other optical super-resolution approaches, e.g., ExM combined with SIM can achieve a lateral resolution of ~25 nm, with a ~60 nm axial resolution [53]. A similar resolution can also be achieved by multiple rounds of expansion [54]. Despite the fact that various protocols are available, the main disadvantage of ExM is that sample preparation is time-consuming and usually requires the optimization of the protocol for each sample type. For a recent review on ExM methods and their applications, see: [55].

## 3. Biological Insights Offered by Nanoscopy

**Figure 4:**
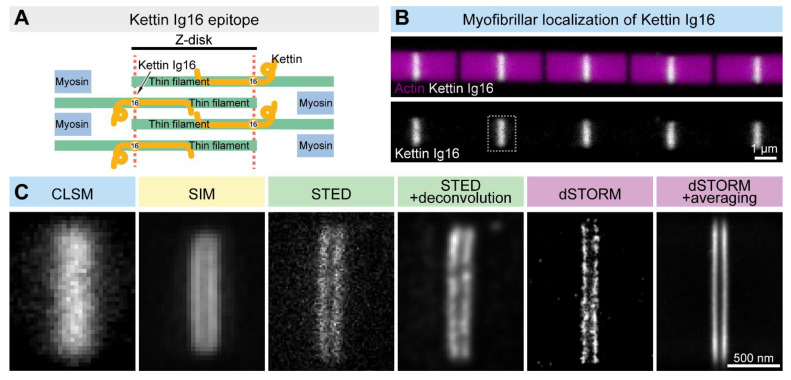
Comparison of the lateral resolution of the different SRM approaches. (**A**) The Kettin Ig16 epitope is found in two stripes separated by ~100 nm in the Z-disk of IFM myofibrils. (**B**) Confocal imaging of the individual myofibrils reveals the Z-disk accumulation of the Kettin Ig16 epitope. The dotted rectangle marks the Z-disk presented in panel C. (**C**) The Kettin Ig16 signal at the Z-disk appears as a single band with confocal microscopy (CLSM), which can be resolved into two individual bands with various super-resolution approaches such as SIM, STED or dSTORM. dSTORM combined with structure averaging (on the right) can produce an image with the most outstanding resolution.

As it is outlined above, despite the rather different rational designs, each SRM technique is suitable to break the resolution limit. Nevertheless, in large part owing to the differences in their technical concept, each method has its own benefits and drawbacks, which are summarized in Table 1, together with the main technical features. The choice of method in the case of a biological sample of interest must take into account these differences, and ideally, it is advised to compare these approaches experimentally. For example, when we decided to study the sarcomeric distribution of a set of actin regulatory proteins, the *Drosophila* Titin ortholog (Kettin) was used as a ‘resolution’ control. Based on immunogold EM images, the 16th immunoglobulin domain of Kettin (Ig16) is present in two stripes at the edges of the Z-disk separated by ~100 nm [56]. Due to their close proximity, the individual ‘lines’ cannot be resolved as distinct structures with confocal laser scanning microscopes (Figure 4). However, SIM or STED microscopy already provides significant improvements, while STORM is able to resolve the sarcomeric structures with a high resolution, which can be further improved by structure averaging (Figure 4). Thus, Z-disk localization of Kettin not only perfectly demonstrated the resolving power of the different nanoscopic approaches but also provided us with a useful batch of information on the available techniques, which was key to our subsequent studies on sarcomere organization. This example will be reviewed, in detail, in the following sections together with several other *Drosophila* models highlighting how resolution improvement can lead to major discoveries in various fields of life sciences. 

## 4. Chemical Synapses

**Figure 5:**
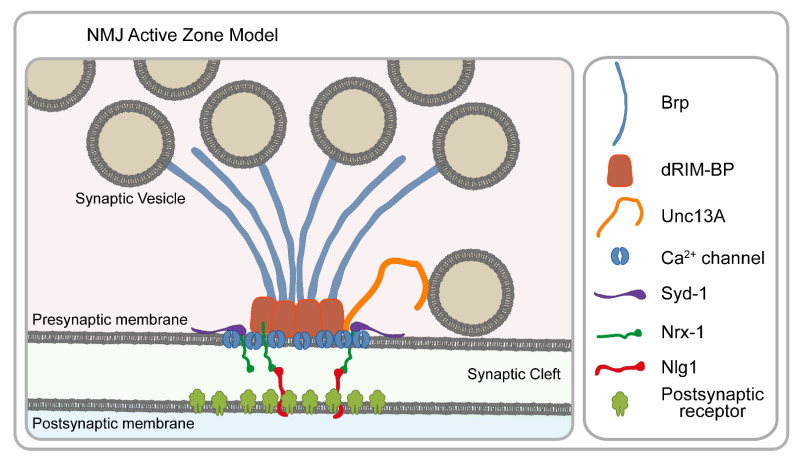
Schematic model of the presynaptic active zone of Drosophila NMJs. The molecular components shown here are involved in the recruitment and spatial arrangement of Ca^2+^ channels (Brp and dRIM-BP), the regulation of release sites (Unc13A and Unc13B) and the initial assembly of AZs (Syd-1, Nrx-1 and Nlg-1). Note that not all AZ-specific proteins are displayed. See text for details.

With the advent of nanoscopic approaches, the glutamatergic larval neuromuscular junction (NMJ) synapses soon became arguably the most successful *Drosophila* model to reveal the molecular architecture of a highly conserved structure with previously unprecedented resolution. Combining STED microscopy with genetic assays and electrophysiology has revealed the nanoscale arrangement and functional roles of the active zone (AZ) proteins (e.g., Brp and dRIM-BP) involved in the recruitment and spatial arrangement of Ca^2+^ channels [24,25], the regulation of release sites (e.g., Unc13A and Unc13B) [57,58,59] and the factors regulating AZ assembly (e.g., Syd-1, Nrx-1 and Nlg-1) [60,61,62] (Figure 5).

The functioning of the neuronal networks relies on the release of synaptic vesicles (SVs) at the AZs. The regulation of synaptic communication is tightly linked to the molecular arrangement of the AZs, which strongly motivated morphological studies, originally restricted to EM. While the basic structural design of synapses was defined by EM, knowledge about their molecular organization was long missing until nanoscopic methods finally allowed resolving it. Pioneering work in the Sigrist lab has established that Bruchpilot (Brp) is a major structural and functional component of the AZ cytomatrix, and although in conventional micrographs, the distribution of Brp appeared as diffraction-limited spots, STED microscopy revealed that Brp forms a doughnut-shaped circular structure at the AZ [25,63], and it is an intrinsic component of the synapse’s electron-dense T-bar structure [24,63]. Later studies revealed that Brp is a filamentous protein with a polarized orientation where the N-terminus faces the presynaptic membrane, while the C-terminus extends into the cytoplasm [24]. Subsequently, it became clear that Brp plays a central role in the organization of the other AZ components including the presynaptic Ca^2+^ channel nanodomains [24]; Brp is also important for SV recruitment [64] and for coupling the AZ to the presynaptic cytoskeleton through regulating the association with Futsch and DAAM [65,66]. In addition to Brp, STED microscopy was key to connect another important structural component to the AZ core, dRIM-BP, which overlays with the Ca^2+^ channels, thereby coupling SV release to the Ca^2+^ channel nanodomains [67]. Parallel to these studies, live super-resolution imaging using *Drosophila* larvae started to explore the assembly mechanisms of the AZ scaffold. New AZs are initiated by the presynaptic Syd-1 accumulation [68]. Syd-1 binds the C-terminus of Nrx-1, which in turn regulates clustering of the postsynaptic Nlg-1 [60]. The pre- and postsynaptic assembly steps are temporally coupled, and there is a feedback mechanism in both directions. By competing with Syd-1 for Nrx-1 binding, Spn (Spinophilin) negatively regulates and fine-tunes the final size of the AZ scaffold [60].

Beyond the remarkable progress that has been made concerning the basic structural organization of the AZs, nanoscopy has also been instrumental to better understand the mechanisms of SV release, AZ remodeling and synaptic plasticity. The efficacy of SV release depends on the coupling distance, defined as the physical distance between the SV and the Ca^2+^ channels. It has been found that the coupling distance in *Drosophila* is determined by targeting two Unc13 isoforms to the presynaptic AZs. Of these, Unc13B is localized 120 nm away from the Ca^2+^ channels, whereas Unc13A is localized to the same position as docked SVs at a 50–70 nm distance from the AZ center [57]. Unc13A is precisely positioned at well-defined sub-AZ positions which is critical for release site generation as well as ensuring short-term synaptic plasticity [57,59]. Perhaps most strikingly, the resolution power of STED is sufficient to detect even slight alterations in the AZ number, size and organization, leading to the demonstration that synaptic plasticity is, at least in part, achieved through rapid remodeling of the AZ scaffolding proteins and that of Unc13 [58]. 

STED microscopy paved the way to these discoveries due to a combination of its favorable properties including compatibility with thicker specimens and live imaging. Nevertheless, STORM microscopy also had a significant contribution by revealing quantitative information on the number of endogenous Brp proteins at NMJ AZs [69]. Since STORM is a single-molecule technique, after careful optimization and validation of the labeling steps, it can provide estimates of the protein copy number. While the AZ architecture clearly exhibits some tissue, developmental state and species-specific variations, the major organization mechanisms appear evolutionarily highly conserved; therefore, it is warranted that the discoveries made in *Drosophila* NMJs will play a pivotal role in understanding the basic principles of neuronal communication.

## 5. Membrane-Associated Periodic Actin Skeleton

**Figure 6:**
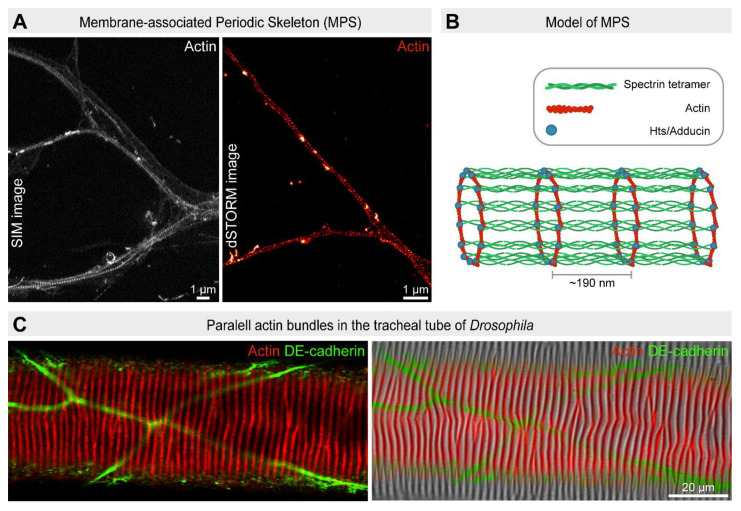
Structure and organization of the membrane-associated periodic actin skeleton. (**A**) Periodic actin rings revealed by SIM and dSTORM microscopy in *Drosophila* primary neuronal cell cultures (12 days in vitro). F-actin is labeled with phalloidin. Scale bar: 1 µm. (**B**) Model depicts the axonal actin rings composed of hts/adducin-capped short actin filaments evenly spaced by spectrin tetramers with a periodicity of ~190 nm. (**C**) Confocal image of a wild-type larval tracheal tube where actin (in red) is organized into parallel running bundles that are perpendicular to the tube axis. The number and phasing of these actin bundles correspond to the taenidial fold pattern displayed on the tracheal cuticle (on the right). DE-cadherin (in green) labels the cell borders.

One of the most recognized early demonstrations of the biological application of SRM was the discovery of periodic axonal actin rings by STORM [70] and later by STED [71,72] and SIM [73] microscopy. Subsequent studies described actin rings in dendrites as well, and a 2D polygonal lattice structure in the soma and dendrites of neurons [74]. This delicate actin-based cortical structure is now referred to as the membrane-associated periodic skeleton (MPS). At a molecular level, the MPS is composed of short actin filaments capped by Adducin/Hts and arranged into ring-like structures underneath the axonal membranes. These rings are connected by spectrin tetramer spacers that maintain a regular ~180 to 190 nm periodicity [70]. This structure is evolutionarily conserved and observed across diverse animal species including *Drosophila* [73,75] (Figure 6) and present in all neuronal cell types examined [75,76]. The genetic tools available in *Drosophila* primary neuronal cell cultures [77], combined with the fast and robust imaging of SIM microscopy, identified numerous factors including components of the Arp2/3 complex and the formin DAAM, required for the nucleation and elongation of the short actin cables of the MPS [73]. However, the biological relevance and dynamics of MPS are still under active investigation and the advantageous characteristics of *Drosophila* models combined with nanoscopy could advance further discoveries. Curiously, periodically arranged actin rings in tubular organs were discovered first in the *Drosophila* tracheal system [78] well before SRM became broadly available. As actin cable periodicity in the embryonic tracheal tubes is in the range of 300–500 nm, the resolution provided by confocal scanning microscopy was sufficient to detect these structures, and phenotypic analysis was a powerful tool to reveal a role in mechanical support and tube diameter maintenance. Taken together with the neuronal MPS data, it appears that the employment of periodically spaced actin rings is a universal theme in living organisms for the construction of thin, flexible tubular structures with a constant diameter. Interestingly, whereas ring periodicity in neurites is determined by spectrin tetramers, the insect tracheal tubes do not seem to rely on that system. Thus, careful revisiting of the *Drosophila* tracheal system with super-resolution methods might occur as an exciting journey in this field. 

## 6. Nanoscopic Reconstruction of Protein Complexes with Structural Symmetry

Macromolecular complexes with well-defined symmetry offer the possibility to obtain many super-resolution images and generate nanoscale reconstructions through particle or structure averaging. The leading example of this approach was demonstrated on nuclear pore complexes (NPCs). Averaged images acquired by dSTORM imaging of NPCs isolated from *Xenopus laevis* oocyte nuclear envelopes revealed the diameter of the central NPC channel and showed the eightfold radial symmetry of the integral membrane protein gp210 [79]. A subsequent study capitalized on this approach, and using the internal rotational symmetry of the NPC, it performed the co-alignment of thousands of dSTORM images to determine the radial position of seven nucleoporin components with sub-nanometer precision within mammalian nuclei [80]. The achieved precision and accuracy of the reconstruction were sufficient to determine the 3D orientation of the Nup107-160 subcomplex, which is a primary component of the NPC scaffold composed of nine Nup subunits, and remarkably, it was also possible to precisely place the subcomplex into the available NPC cryo-EM density map [80]. This example highlighted that nanoscopy combined with structure averaging approaches can bridge the gap between the atomic resolution methods and conventional light microscopic methods. A similar methodology was later applied to address structural and biological questions of various symmetric structures including the centrosomes [8], the synaptonemal complex [81], the endocytic machinery [82] and the sarcomeric complexes [50] in multiple model systems, including *Drosophila*.

## 7. Centrosome

The fundamental microtubule organizing centers of animal cells are the centrosomes, which are non-membrane-bound organelles composed of centrioles (also serving as basal bodies for cilia), and the surrounding pericentriolar material (PCM) [9,83]. The centrosome fulfills diverse roles including the regulation of spindle assembly, cytokinesis and cilia formation, and it is also a docking and signaling station controlling the microtubule-mediated transport of numerous proteins. PCM directly anchors the microtubule minus ends through the γ-tubulin ring complexes (γTRCs), and it coordinates centriole duplication during the cell cycle. The diameter of the centriole is around 200–250 nm; therefore, it is at the resolution limit of conventional light microscopy. 

**Figure 7:**
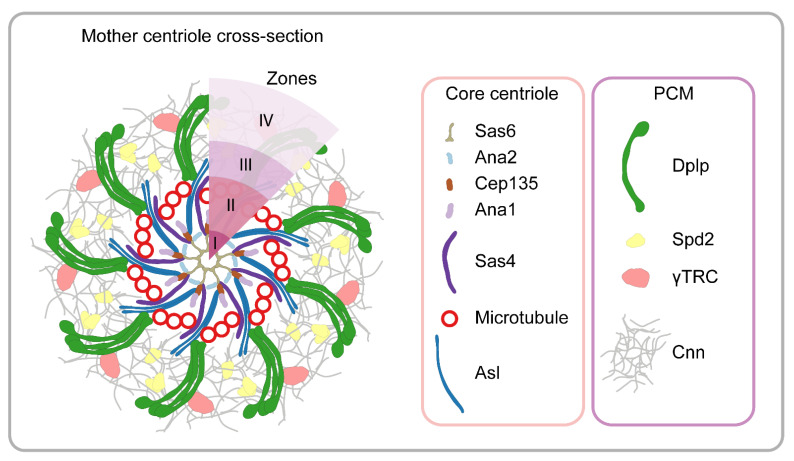
Schematic cross-sectional model of the mother centriole and the associated pericentriolar material at the point of mitotic entry. The molecular components of *Drosophila* centrosomes are organized into five zones defined by super-resolution microscopy. Note that not all centrosome-specific proteins are displayed. Additionally, note that zone V at the distal tip of the centriole is not displayed in the cross-sectional view. See text for details.

Previous structural studies with EM revealed the highly ordered cylindrical-shaped ninefold symmetrical structure of the centrioles and suggested an amorphous PCM organization based on the observed homogeneous electron density. In *Drosophila*, classical genetic screens and genome-wide RNAi approaches have identified numerous centrosome components [84,85]. The first demonstration of the power of nanoscopy in this field was when 3D SIM resolved the fine structure of the PCM, thereby challenging the long-standing view of amorph organization [8]. By exploiting the regular and symmetric nature of these organelles, a high-resolution molecular model was built using averaging and cross-correlation, which ultimately revealed the layered organization of the PCM [8]. Following the initial observations, a number of studies demonstrated that the *Drosophila* centrosome is composed of five layers or zones (Figure 7) (for a recent review, see: [86]). Zones I and II constitute the centriole, zones III and IV form the surrounding PCM and zone V is at the distal end of the centriole [8,87,88]. Zone I includes cartwheel proteins, e.g., Ana2 and Sas6. Zone II components (e.g., Sas4, Spd2, Polo and MTs) are organized into a toroid shape around the central cartwheel with a diameter under ~200 nm. Cep135, similar to Ana1, is localized between the inner and outer components of the centriole in *Drosophila*, where it maintains their connection and regulates the width and stability of the centriole [88,89]. Zone III (also known as the proximal layer) is composed of proteins involved in centrosome maturation (e.g., Asl and Dplp). 

A few components of the centrosome are elongated and span across multiple zones; however, specific labeling of their termini not only helped their precise positioning but also helped to reveal their orientation. For example, the C-terminus of Dplp overlaps with the centriolar microtubule wall, while its N-terminus is extended into the PCM, where it is essential for the initial recruitment of additional PCM components [8]. 

Zone IV is a transient structure that is assembled at the beginning of mitosis. Spd2 and Cnn are recruited there in a mutually dependent manner to form a scaffold structure that expands outwards. Additional components of the PCM, including γ-tubulin, are loaded onto this structure [90,91]. Zone V includes the conserved Cep97 and CP110 proteins that localize to the distal end of centrioles, where they maintain the centriole size and stability and regulate the early steps of cilium biogenesis [92,93].

The 3D SIM method provided significant insights concerning how centrioles are organized and further confirmed that the PCM, which was previously thought to be a fuzzy structure, is, in fact, an ordered assembly of proteins (Figure 7). The higher-order organization of the PCM is an evolutionarily conserved property of centrosomes, and a highly similar arrangement was found in vertebrates [94,95]. 

Unlike SIM, STED and SMLM have been less popular tools even though numerous studies demonstrated that these could resolve clusters of proteins, whereas 3D SIM resolves them as continuous rings [96,97]. In addition, SMLM, combined with particle averaging, can produce multicolor 3D reconstructions from dual-color 2D datasets with outstanding resolution [98]. Due to these potentials, we anticipate that the STED and SMLM approaches will be put to work for further clarification of the centrosome architecture and dynamics. 

## 8. Sarcomeric Assemblies

**Figure 8:**
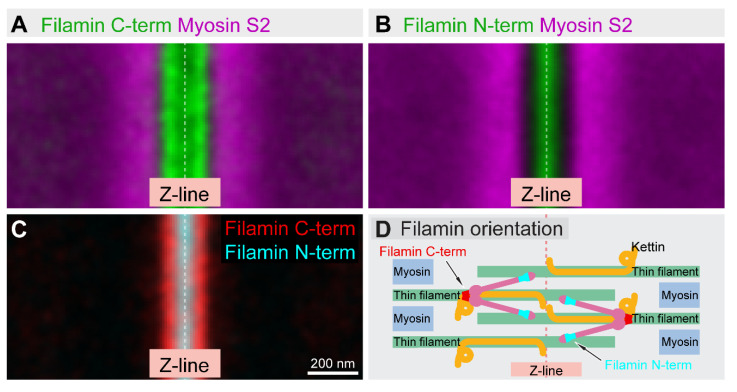
Sarcomeric localization and orientation of Filamin revealed by dSTORM microscopy. (**A**) A Filamin C-terminus-specific antibody revealed a double-line distribution along the edges of the Z-disk separated by roughly 100 nm. (**B**) In contrast to this, the N-terminus-specific antibody displayed a ‘band-type’ distribution in the middle of the Z-disk. (**C**) The aligned representation clearly demonstrates that we can distinguish between the two epitopes, and we can unambiguously determine the orientation of the molecule within the Z-disk. (**D**) Schematic shows the position and orientation of the Filamin dimers in the Z-disk of *Drosophila* IFM sarcomeres.

Sarcomeres represent one of the most highly ordered macromolecular assemblies where different EM methods have already produced quasi-atomic resolution models for the contractile actin–myosin complexes found in the thin and thick filament overlap regions (A-bands) [99,100]. Despite the wealth of information collected, the precise molecular architecture of the Z-disk-containing I-band (devoid of myosin filaments) and the M-line-containing H-zone (devoid of actin filaments) is much less well understood. These regions are the distinguishing features of sarcomeres that regulate the assembly and dynamics of both the thick and thin filaments; therefore, determining their precise molecular organization is indispensable in order to understand the molecular mechanisms of sarcomere assembly and dynamics. The indirect flight muscle (IFM) of *Drosophila* is a uniquely suitable model system to study the assembly and growth of myofibrils since the function of the IFM is not needed for viability and can be easily assayed by testing for flight. Therefore, genetic analysis is suitable for functional studies, while the isolated individual myofibrils are convenient for microscopic analysis as they are relatively thin and lay flat on the coverslip. The isolated myofibrils are parallel to the focal plane, and the acquired 2D projections of the sarcomeres reveal the lateral distribution of proteins at the H-zones and I-bands. Based on the epitope distributions along the longitudinal axis of the myofibrils, the acquired patterns can be classified into multiple categories, and by rotation and translation, the localization information from hundreds of sarcomeres can be co-aligned and averaged. After applying structure alignment, the average position of a fluorescent label can be determined with a standard deviation of around 5 to 10 nm, along the longitudinal axis of the myofibril. We used this approach to generate a localization atlas characterizing the precise sarcomeric position of 14 epitopes in the H-zone and 21 epitopes in the I-band [50,101,102]. The analysis established the position of the key sarcomeric landmarks, including the (+) and (−) ends of the thin filaments, ends of the thick filaments and, thus, the widths of the H-zone, the I-band and the Z-disk. Based on measurements, the position and orientation of the Tropomyosin–Troponin complex on thin filaments could be determined [50]. Furthermore, we revealed and validated many of the previously established molecular arrangements, including the orientation of some of the large modular proteins such as the H-zone organizer Obscurin or the I-band elastic proteins Kettin and Projectin [50]. The nanoscopic topology of the Z-disk suggested that Zasp52 is a core component, which is in an ideal position to regulate the incorporation and position of the crosslinking α-actinin molecules accumulating into two bands separated by ~80 nm. The determined position and orientation of the actin binding Filamin dimer identified it as part of the C4 EM density, which connects the parallel thin filaments at the edge of the Z-disk (Figure 8). Additionally, Filamin is connected to Kettin, and based on nanoscopic measurements, the site of the interaction could be narrowed down to a specific region of the protein [50]. In contrast to the (+) end-dependent actin polymerization in non-muscle cells, actin filaments in striated muscles somehow elongate from their (−) ends (facing the sarcomeric H-zones) with a virtually unknown mechanism. Examination of the nanoscale distribution of the actin regulatory proteins linked to (−) end elongation led to the unexpected finding that these proteins are organized into two distinct spatial domains. The growth-promoting SALS and the elongation-blocking Tmod are both localized right to the pointed end of the thin filaments, while the well-known formin type of actin assembly factors (DAAM and Fhos) and the G-actin binding profilin occupy a more central location about 20–30 nm away [50]. Due to physical separation of the pointed ends from the actin assembly machinery, these results do not conform with models based on actin monomer incorporation at the pointed end; they rather support the possibility of formin-mediated F-actin oligomer formation in the central zone that could subsequently fuse to the pointed ends under the control of Tmod and SALS.

Beyond the mere protein distribution analysis, the sarcomeric localization information could be used as physical constraints that, combined with the results of structural, biochemical and genetic studies, allow the generation of a refined molecular model of the sarcomeric H-zones and I-bands. Whereas there is no doubt that these models need further refinements, it certainly helps to comprehend the scale of resolution available with this approach, and with its predictive power, we hope that it will pave the way to further mechanistic and structural modeling of the sarcomere.

## 9. Concluding Remarks and Future Perspectives

The resolution revolution of the last decade pushed fluorescent microscopy closer to the ultimate goal of molecular-scale resolution. It is now obvious that the next barrier that we need to overcome stems from the fact that all fluorescent microscopic techniques detect the labeling molecules and not directly the molecules of interest. Consequently, even the methods providing the highest theoretical resolution are limited by multiple aspects of labeling [103]. For example, sparsely labeled structures cannot be clearly resolved, and the size of the fluorescent label also becomes significant at high resolution. With indirect immunochemistry, primary and secondary antibodies increase the apparent size of the visualized structure and might introduce a localization bias of about 10–20 nm. Furthermore, antibody penetration and/or epitope accessibility can be limited in multiple tissues due to the size of the antibodies. Therefore, it seems urgently important to improve the labeling methods to ensure high-affinity labeling of precisely mapped epitopes with fluorophores of small size and high brightness/photostability. One strategy is to use genetically encoded tags, and for this, genome-wide resources are already available in *Drosophila* [104]. This approach is reasonably efficient and solves some of the above-outlined problems. However, the frequently used protein tags are still quite large, and many of the tagged proteins are non-functional. An emerging alternative is to use single-domain antibodies, also known as nanobodies. Nanobodies can bind to their targets with high affinity, often in the low nanomolar range [105]. The ‘linkage error’ can also be significantly reduced by the use of labeled nanobodies, which are considerably smaller (~2.5 nm) than the standard antibodies [106], and unlike the conventional antibodies, isolated nanobodies can be readily expressed in bacteria, which ensures a stable quality and reproducibility. Provided that the development of the new fluorescent dyes and labeling methods will be nearly as fast as the advance of the microscopy technologies in the past decade, super-resolution imaging will certainly remain one of the most fascinating and robust approaches for cellular and structural studies in the near future.

## Figures and Tables

**Table 1:** Comparison of the different SRM techniques. *: low, **: medium, ***: high.

	SIM	STED	STORM/PALM	ExM
Concept to overcome the resolution limit	High-frequency information containing interference generated by patterned illumination	Stimulated de-excitation is used to produce a narrower emission zone	Fluorophores are modulated in the time scale to separate and localize them one by one	Isotropic sample expansion is used to increase the distance between the molecules
Microscopy type	Widefield	Laser scanning confocal	Widefield	Widefield/laser scanning confocal/spinning disk confocal
Lateral resolution	~100 nm (linear) ~50 nm (nonlinear)	~30–50 nm	~20 nm	~70 nm ~25 nm (with SIM)
Axial resolution	~300 nm (linear) ~120 nm (nonlinear)	~30 nm	~50 nm	~200 nm ~60 nm (with SIM)
Fluorophore type	Conventional fluorescent proteins and dyes	Photostable dyes and fluorescent proteins	STORM: photoswitchable dyes PALM: photoswitchable fluorescent proteins/dyes	Special labeling probe (able to survive the homogenization)
Phototoxicity	*/**	**/***	**	-
Photobleaching	**/***	**/***	*	*
Live imaging	Well suited	Moderately suited	Limitedly suited	No
Post-image processing	Yes	No	Yes	Yes
Maximum number of simultaneous colors	4	2	2–3	4
Concerns	Out-of-focus signals	Photobleaching	Over/under-labeling artifacts	Time-consuming optimization

## Abbreviations

Ana1	Anastral spindle 1
Ana2	Anastral spindle 2
Arp2/3	Actin-related protein 2/3
Asl	Asterless
AZ	Active zone
Brp	Bruchpilot
Cep97	Centrosomal protein 97 kDa
Cep135	Centrosomal protein 135 kDa
CLSM	Confocal laser scanning microscopy
Cnn	Centrosomin
CP110	Centriolar coiled coil protein 110 kDa
DAAM	Disheveled Associated Activator of Morphogenesis
Dplp	Drosophila pericentrin-like protein
dRIM-BP	Drosophila Rab3-Interacting Molecule-Binding Protein
dSTORM	Direct stochastic optical reconstruction microscopy
EM	Electron microscopy
ExM	Expansion microscopy
F-actin	Filamentous actin
FHOS	Formin homology 2 domain containing
FWHM	Full width at half maximum
G-actin	Globular actin
gp210	Glycoprotein 210
GSD	Ground state depletion
γTRC	γ-tubulin ring complex
Hts	Hu-li tai shao
IFM	Indirect flight muscle
MINFLUX	Minimal photon flux
MPS	Membrane-associated periodic skeleton
MT	Microtubule
Nlg-1	Neuroligin-1
NMJ	Neuromuscular junction
NPC	Nuclear pore complex
Nrx-1	Neurexin-1
Nup	Nucleoporin
PAINT	Point Accumulation for Imaging in Nanoscale Topography
PALM	Photoactivated localization microscopy
PA NL-SIM	Patterned activation nonlinear-structured illumination microscopy
PCM	Pericentriolar material
PSF	Point spread function
SALS	Sarcomere length short
Sas4	Spindle assembly abnormal 4
Sas6	Spindle assembly abnormal 6
SIM	Structured illumination microscopy
SMLM	Single-molecule localization microscopy
Spd2	Spindle defective 2
SPIM	Single-plane illumination microscopy
Spn	Spinophilin
SRM	Super-resolution microscopy
STED	Stimulated emission depletion
STORM	Stochastic optical reconstruction microscopy
SV	Synaptic vesicle
Syd-1	Synapse Defective 1
TIRF	Total internal reflection fluorescence
Tmod	Tropomodulin
Unc13A	Uncoordinated 13A
Unc13B	Uncoordinated 13B
